# High-salt transcription from enzymatically gapped promoters nets higher yields and purity of transcribed RNAs

**DOI:** 10.1093/nar/gkad027

**Published:** 2023-01-31

**Authors:** Kithmie MalagodaPathiranage, Elvan Cavac, Tien-Hao Chen, Bijoyita Roy, Craig T Martin

**Affiliations:** Department of Chemistry, University of Massachusetts Amherst, Amherst, MA 01003, USA; Department of Chemistry, University of Massachusetts Amherst, Amherst, MA 01003, USA; RNA and Genome Editing, New England Biolabs, Beverly, MA 01938, USA; RNA and Genome Editing, New England Biolabs, Beverly, MA 01938, USA; Department of Chemistry, University of Massachusetts Amherst, Amherst, MA 01003, USA

## Abstract

T7 RNA polymerase is commonly used to synthesize large quantities of RNA for a wide variety of applications, from basic science to mRNA therapeutics. This *in vitro* system, while showing high fidelity in many ways, is also well known for producing longer than encoded RNA products, particularly under high-yield reaction conditions. Specifically, the resulting product pool is contaminated by an often disperse collection of longer *cis*-primed extension products. In addition to reducing yield via the conversion of correctly encoded RNA to longer products, self-primed extension generates partially double-stranded RNAs that can trigger the innate immune response. Extensive and low-yield purifications are then required to produce therapeutic RNA. Under high-yield conditions, accumulating concentrations of RNA effectively compete with promoter DNA for polymerase binding, driving self-primed extension at the expense of correct initiation. In the current work, we introduce a simple and novel modification in the DNA to strengthen promoter binding, shifting the balance back toward promoter-driven synthesis and so dramatically reducing self-primed extension. The result is higher yield of the encoded RNA at the outset and reduced need for extensive purifications. The approach can readily be applied to the synthesis of mRNA-length products under high-yield conditions.

## Graphical Abstract

**Graphical Abstract:**
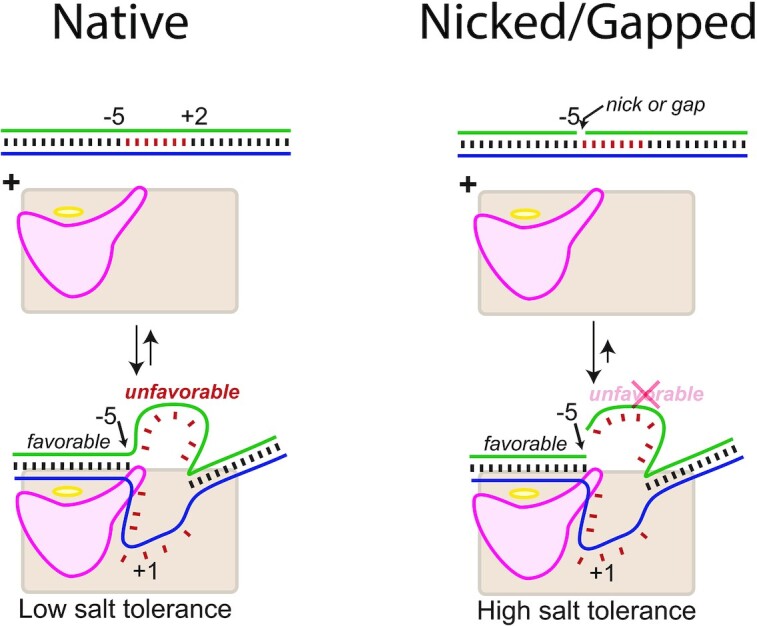
Introduction of a gap in the melted region of the promoter strengthens overall binding, allowing increased salt tolerance.

## INTRODUCTION

From novel vaccine platforms, illustrated by the SARS-CoV-2 mRNA vaccines, to gene therapy, RNA stands poised to play a central role in therapeutics of the future (1–3). Through advances in CRISPR technologies, RNA is also increasingly playing a role in diagnostics (4,5). RNA of all but the shortest lengths is synthesized using the enzyme T7 RNA polymerase (or one of its close relatives), as this enzyme is robust and can yield large quantities of RNA (6,7). However, undesired products often contaminate the desired RNA, in often unpredictable ways (8–10). This is perhaps most impactful currently in the mRNA therapeutics field, where contaminating double-stranded RNAs (dsRNAs) can trigger a potentially deleterious innate immune response (11,12), but contaminants almost certainly impact other studies as well, from basic research in cell and molecular biology or biochemistry and biophysics, to synthetic biology, to RNA nanotechnology and therapeutics.

We and others have shown that these products arise from rebinding of released RNA to the enzyme in a mode that allows its 3′ end to pair with upstream sequences in the RNA and prime extension encoded by the RNA itself (8–10). Under high-yield reaction conditions, this secondary reaction can become dominant. Extensive rebinding of correct length RNA to prime extension not only generates double-stranded impurities, but also reduces the yield of the correct product by consuming it. The resulting impure mixture requires chromatographic (or gel) purifications that further reduce yields and are imperfect—separating a 2050-nucleotide RNA from an encoded 2000-nucleotide RNA is generally beyond these approaches (13–15).

Rebinding of the product RNA to the RNA polymerase should involve (at least) nonspecific, electrostatic interactions. Thus, one would expect that increasing ionic strength should reduce rebinding, and therefore reduced self-primed extension. We have observed that elongation complexes of T7 RNA polymerase are relatively tolerant to moderate salt concentrations (16), but initial promoter binding by T7 RNA polymerase is sensitive to ionic strength, reducing the general utility of transcription at high salt.

**Figure 1.**
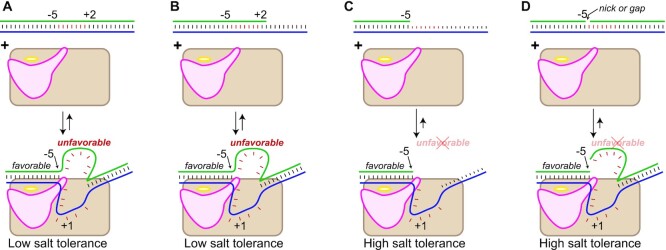
Schematic of promoter binding by T7 RNA polymerase. (**A**) In fully duplex DNA, energetically favorable duplex interactions upstream of and including position −5 are used to drive the unfavorable melting of bases from position −4 to ∼+3. (**B**) Partially single-stranded duplex up to +2 shows weaker binding due to unfavorable melting of bases in the melting region from position −4 to ∼+2. (**C**) Removal of the unfavorable melting requirement decreases net melting energetics. (**D**) Nicking (or gapping) the DNA as shown decouples these processes and removes entropic constraints that contribute to the melting barrier. Stronger binding in panels (C) and (D) allows increased tolerance to destabilization induced by increasing ionic strength.

As illustrated in Figure 1, we (and others) have reported that in promoter binding, T7 RNA polymerase uses some of the energy derived from formation of duplex promoter contacts to drive melting of the DNA near the transcription start site (17–20). DNA constructs lacking a part of the nontemplate strand in this melting region (partially single stranded) bind to the enzyme with substantially greater affinity than do the fully duplex constructs.

In this work, we demonstrate that enhancement of promoter binding using partially single-stranded or nicked/gapped promoter constructs yields initiation complexes that are more tolerant to increasing ionic strength. Transcription under optimized salt concentrations allows efficient initiation, while inhibiting the product rebinding that leads to self-primed extension. We further describe a novel approach toward generating uniquely gapped promoter constructs in long double-stranded DNA templates. Together, these approaches allow RNA synthesis with a dramatic reduction in self-primed extension (higher purity of the encoded RNA). Since primed extension derives from correct length RNA, the improved purity is accompanied by an overall increase in yield of the desired RNA.

## MATERIALS AND METHODS

### Enzyme and reagents

His-tagged T7 RNA polymerase was prepared from *Escherichia coli* strain BL21 carrying the plasmid pBH161, purified and characterized as previously described (21).

### DNA constructs

**Figure 2.**
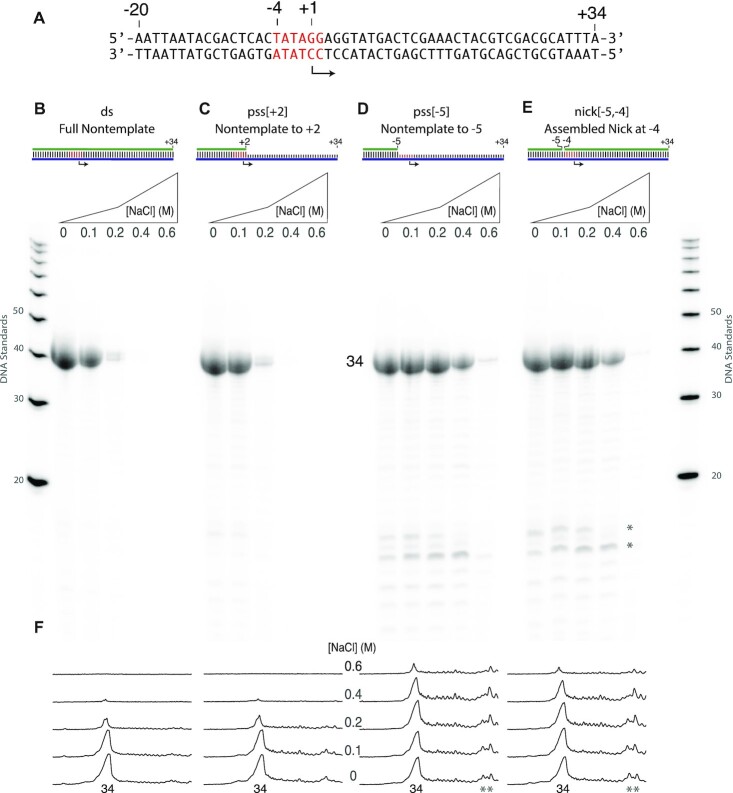
Stronger promoter binding confers salt tolerance. (**A**) A DNA template strand encoding a runoff 34mer RNA known to produce relatively low amounts of self-primed extension was paired with different nontemplate DNA strands to generate constructs with native melting requirements [(**B**) ds and (**C**) pss[+2]] and relaxed melting requirements [(**D**) pss[−5] and (**E**) nick[−5, −4]]. Experiments in panels (B)–(E) drive high-yield synthesis under conditions of increasing added NaCl, as shown. Transcription reactions contained 1.0 μM DNA and 1.0 μM RNA polymerase and were incubated at 37°C for 4 h, under conditions described in the ‘Materials and Methods’ section. The individual lane tracings in panel (**F**) quantify retention of transcription at high added salt for the relaxed melting constructs. See Supplementary Figure S2 for replicates.

**Figure 3.**
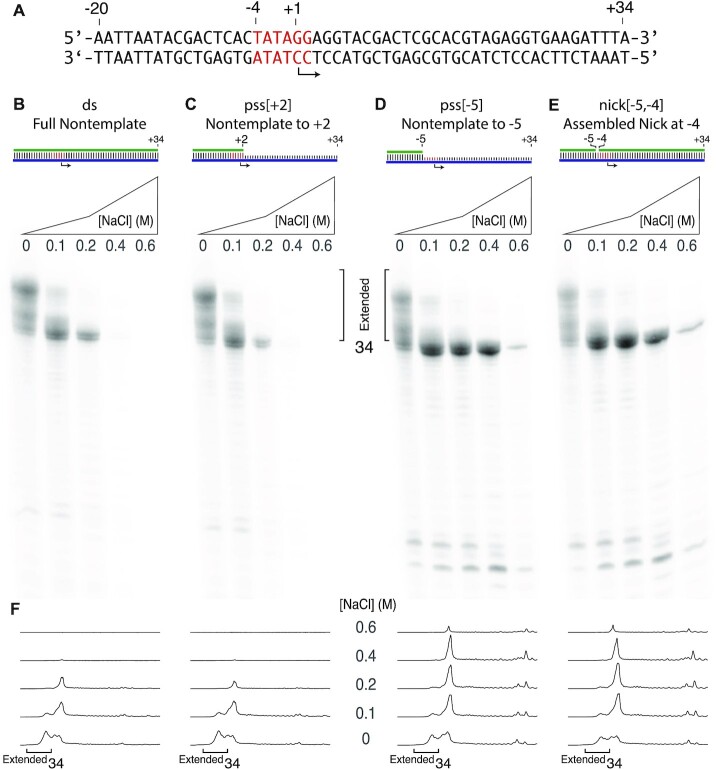
Added NaCl reduces self-primed extension products. (**A**) Template DNA encoding a runoff 34mer RNA known to prime extension was paired with nontemplate DNA strands (**B**–**E**) and transcribed as in Figure 2. The tracings in panel (**F**) quantify retention of transcription at high added salt for the relaxed melting constructs. Reaction conditions were as in Figure 2. See Supplementary Figure S3 for replicates.

**Figure 4.**
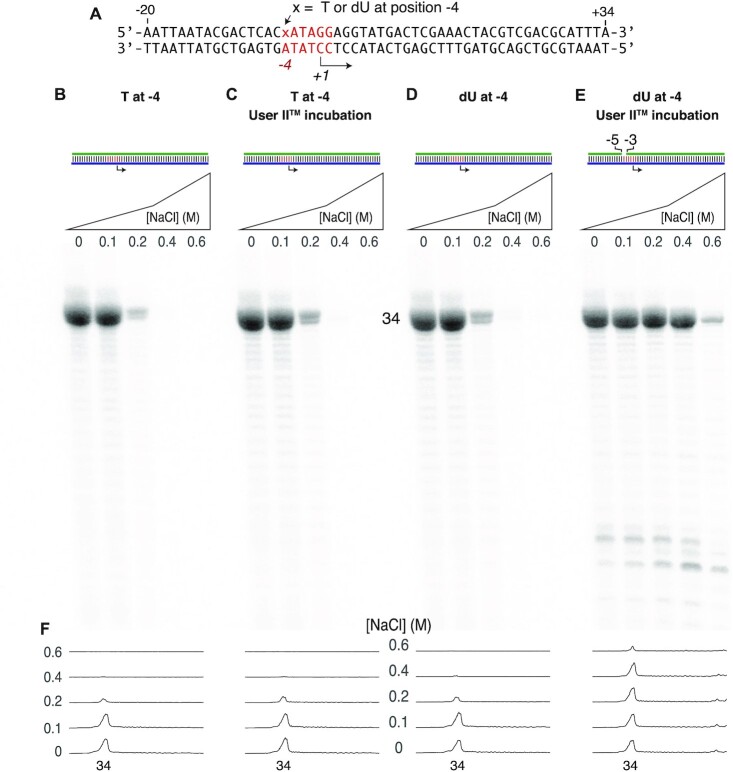
Targeted enzymatic gapping increases salt tolerance. (**A**) Template DNA encoding the runoff 34mer RNA of Figure 2 was paired with different nontemplate DNA strands: panels (**B**) and (**C**) have a native T at position −4 of the nontemplate strand, while panels (**D**) and (**E**) have dU at that position. Transcription reactions contained 1.0 μM DNA and 1.0 μM RNA polymerase and were incubated at 37°C for 4 h, under conditions described in the ‘Materials and Methods’ section. As expected, only the enzymatic excision of dU from position −4 of the nontemplate strand (E) yields salt tolerance. Individual lane tracings are shown in panel (**F**). Reaction conditions were as in Figure 2. See Supplementary Figure S4 for replicates.

**Figure 5.**
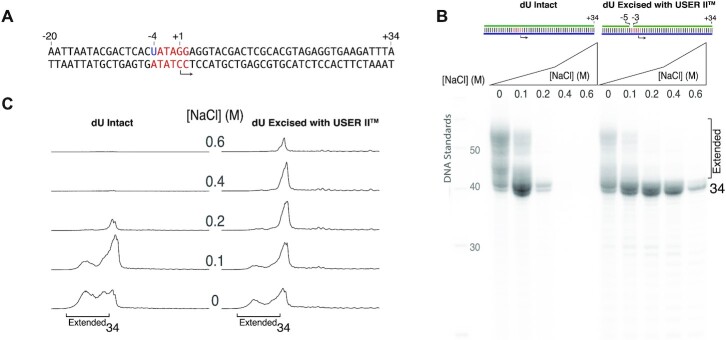
A targeted gap plus high-salt inhibition of self-primed extension. (**A**) Doubled-stranded DNA encoding the runoff 34mer RNA from Figure 3 and containing dU at position −4 was transcribed directly (unexcised) or following excision of dU with the USER^®^ II enzyme generates a gap. (**B**) Transcription reactions contained 1.0 μM DNA and 1.0 μM RNA polymerase and were incubated at 37°C for 4 h, under conditions described in the ‘Materials and Methods’ section. Quantification (**C**) demonstrates results similar to those of Figure 3. Reaction conditions were as in Figure 2. See Supplementary Figure S5 for replicates.

**Figure 6.**
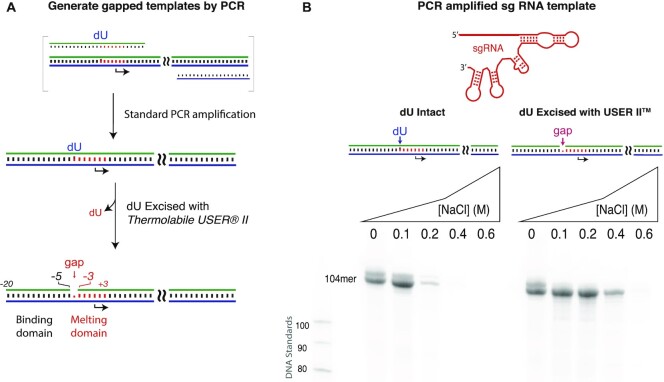
Extension to a 104-nucleotide sgRNA. (**A**) Schematic diagram of template preparation of DNA template with PCR, incorporating dU via the upstream PCR primer. (**B**) Synthesis of a 104-nucleotide guide RNA from DNA containing intact or excised dU at position −4, under elevated salt concentrations. Transcription reactions contained 0.125 μM DNA and 0.125 μM RNA polymerase and were incubated at 37°C for 4 h, under conditions described in the ‘Materials and Methods’ section. See Supplementary Figure S6 for replicates.

**Figure 7.**
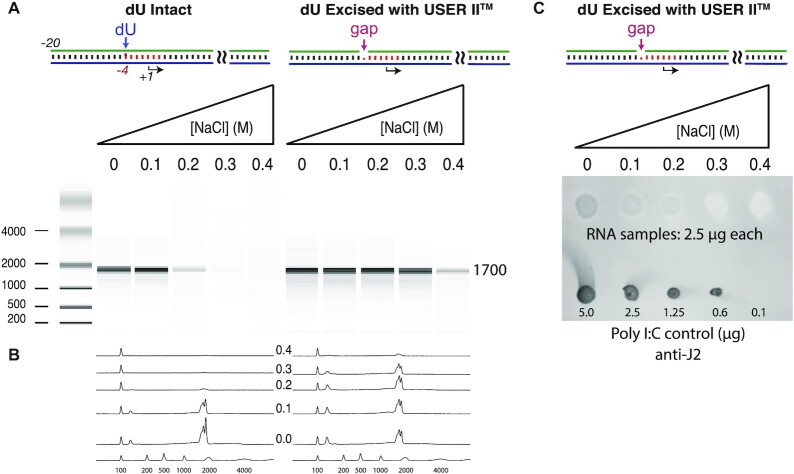
Extension to a 1700-nucleotide mRNA. The approach in Figure 6 was extended to a 1700-nucleotide mRNA. (**A**) Transcription of (M2) DNA containing intact or excised dU with elevated salt conditions. (**B**) Quantitative traces of panel (A). (**C**) Immunoblot of mRNA transcribed with dU-excised template, under increasing added salt concentrations. Transcription reactions contained 0.035 μM (M2) DNA and 0.035 μM RNA polymerase and were incubated at 37°C for 1 h, under conditions described in the ‘Materials and Methods’ section. See Supplementary Figure S7 for replicates.

**Figure 8.**
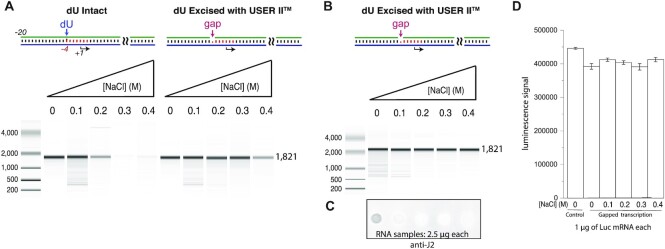
mRNA generated from the gapped system is low immunogenic and functional. (**A**) Transcription of (M3) DNA, incorporating CleanCap AG (TriLink) and encoding a poly-A tail, using DNA templates containing intact or excised dU, to generate a gap at position −4 of the promoter, as indicated. Transcription reactions contained 0.035 μM (M3) DNA and 0.035 μM RNA polymerase and were incubated at 37°C for 1 h, as described in the ‘Materials and Methods’ section. Equal amounts of mRNA synthesized from the dU-excised promoter were analyzed by gel (**B**) and probed by immunoblot (**C**), as in Figure 7C. (**D**) Luciferase activity was measured after transfection of equal amounts (1.0 μg) of mRNA synthesized from the enzymatically gapped promoter (*N* = 3), indicating functional mRNAs. See Supplementary Figure S8 for replicates.

DNA oligonucleotides used as transcription templates were purchased from Integrated DNA Technologies; sequences are shown in Supplementary Table S1. For experiments in Figures 2 and 3, equimolar amounts of the indicated DNA strands were mixed to assemble the functional DNA, as shown. For experiments in Figures 4 and 5, fully duplex oligonucleotide constructs were similarly assembled, containing T or dU at position −4, as indicated. For experiments in Figures 6–8 and Supplementary Figures S6–S9, template DNA was PCR amplified with Phusion U DNA polymerase, using an upstream primer containing T or dU at position −4, and was then cleaned up with Qiagen PCR cleanup kit. In Figures 4–8 and Supplementary Figures S4–S9, aliquots were then incubated with the Thermolabile USER^®^ (Uracil-Specific Excision Reagent) II Enzyme (New England Biolabs) system to excise dU, as indicated in the figures, to introduce a gap in the nontemplate strand. USER^®^ II excision reactions for oligonucleotide templates contained 10 μM duplex DNA, excision reactions for 104-nucleotide-long guide RNA templates contained 1.25 μM DNA and excision reactions for longer (mRNA-length) RNA templates contained 0.35 μM DNA. In each, the DNA was dissolved in NEB CutSmart^®^ buffer with 20 U/ml of Thermolabile USER^®^ II Enzyme system. After 1 h incubation at 37°C, the reactions were heat inactivated at 65°C for 10 min.

### Transcription reactions generating short RNA

High-yield transcription reaction contained 1 μM of the indicated DNA, 1 μM T7 RNA polymerase, and 7.5 mM each of guanosine triphosphate (GTP), cytidine triphosphate (CTP), adenosine triphosphate (ATP) and uridine triphosphate (UTP). Reactions were carried out at 37°C for 4 h in a buffer containing 40 mM magnesium acetate, 30 mM 4-(2-hydroxyethyl)-1-piperazineethanesulfonic acid (HEPES), 25 mM potassium glutamate, 0.25 mM ethylenediaminetetraacetic acid (EDTA) and 0.05% Tween 20 and were supplemented with 2000 U/ml of RNase Inhibitor, Murine (New England Biolabs) and 5 U/ml of inorganic pyrophosphatase (yeast, New England Biolabs).

### Transcription reactions generating guide RNA

High-yield transcription reaction contained 0.125 μM of the indicated DNA, 0.125 μM T7 RNA polymerase, and 7.5 mM each of GTP, CTP, ATP and UTP. Reactions were carried out at 37°C for 4 h in a buffer containing 40 mM magnesium acetate, 30 mM HEPES, 25 mM potassium glutamate, 0.25 mM EDTA and 0.05% Tween 20 and were supplemented with 2000 U/ml of RNase Inhibitor, Murine (New England Biolabs) and 5 U/ml of inorganic pyrophosphatase (yeast, New England Biolabs).

### Transcription reactions generating longer RNA

Transcription reactions contained 0.035 μM of the indicated DNA, 0.035 μM T7 RNA polymerase, 5 mM of each of GTP, CTP, ATP and UTP, and 4 mM of CleanCap AG (TriLink), where indicated. Reactions were carried out at 37°C for 1 h in a buffer containing 30 mM magnesium acetate, 30 mM HEPES, 25 mM potassium glutamate, 0.25 mM EDTA and 0.05% Tween 20 supplemented with 2000 U/ml of RNase Inhibitor, Murine (New England Biolabs) and 5 U/ml of inorganic pyrophosphatase (yeast) and were stopped by heat inactivation at 70°C for 5 min. The reactions were treated with TURBO™ DNase I (Invitrogen) to remove the template and then cleaned up with Monarch RNA Cleanup Kit (New England Biolabs).

### Detection of dsRNA contamination

Immunoblot analysis was carried out by spotting purified mRNA on a positively charged Nylon membrane (Nytran SC, Sigma–Aldrich), blocking the membrane with 5% (w/v) nonfat dried milk in TBS-T buffer [20 mM Tris, pH 7.4, 150 mM NaCl, 0.1% (v/v) Tween 20] and probing it with anti-dsRNA antibody (clone rJ2 1:5000; Scicons) at 4°C overnight. Immunoblots were probed with IRDye™ 680 or 800 conjugated secondary antibodies (Cell Signaling Technologies) for 1 h and imaged with a Typhoon FLA 9500 imager to determine dsRNA contamination. The amount of RNA analyzed is indicated in the figures. Poly I:C was used as a dsRNA control for the immunoblots.

### Luciferase assay

Synthesized, purified mRNAs were transfected into human embryonic kidney cells (HEK 293) using the TransIT-mRNA Transfection Kit (Mirus Bio). The expression from the luciferase mRNA was analyzed by measuring the luciferase activity from the media measured 6 h post-transfection. The luciferase activity was measured with BioLux Cypridina Luciferase Assay Kit (New England Biolabs) using a Centro LB 960 luminometer (Berthold) in relative light units.

### Gel electrophoretic analyses

Reaction products from short templates and guide RNA were analyzed using 20% polyacrylamide, denaturing (7 M urea) gel electrophoresis. Transcribed RNAs were labeled by including [α-^32^P] ATP (PerkinElmer) in the reaction mixture (without reducing the total concentration of ATP). All gel experiments were repeated at least twice and showed no significant differences. Quantifications of gel data from the Typhoon FLA 9500 laser imager used ImageJ v2.1.0/1.53c (https://imagej.nih.gov/ij/) (22,23) analysis of unprocessed and uncompressed TIF images. The longer RNA was analyzed with an Agilent 2100 Bioanalyzer with the RNA nanochips obtained by Agilent Technologies.

## RESULTS

In this study, we have used sequences previously shown to generate self-primed, partially double-stranded extension products, as well as those shown to produce substantially less extension (10,24,25). We have also generated longer guide RNAs and mRNAs to demonstrate the wide range of applicability of this method. In all, we predict that strengthening promoter binding, as in the constructs of Figure 1, will make promoter-directed transcription more tolerant to salt. To test this initial prediction, we carried out transcription on a DNA sequence that encodes RNA with little apparent self-primed extension.

### Strengthened promoter binding yields salt tolerance

Building on studies noted above, we prepared DNA with native and reduced barriers to promoter melting that are expected to yield native and stronger promoter binding, respectively (18). The prediction that stronger binding will yield increased tolerance to salt is confirmed by the data shown in Figure 2. Constructs with full-length nontemplate (Figure 2B) and with a nontemplate strand ending at +2 (Figure 2C) both require melting from positions −4 to ∼+ 2 and show native (high) sensitivity to added NaCl. In contrast, constructs with the nontemplate strand truncated at position −5 (Figure 2D) do not require promoter melting and transcribe well to at least 0.4 M added NaCl. A construct that is fully double stranded but contains a break (‘nick’) in the nontemplate strand at the upstream edge (between the −4 and −5 bases) of the melting region (Figure 2E) decouples upstream binding energetics from bubble melting and weakens the cooperativity of duplex stability in that region. As expected, transcription from this construct (Figure 2E) shows similar increased tolerance to added NaCl.

Quantification of gel band intensities shown in Figure 2F confirms that transcription from the constructs lacking promoter melting is tolerant to at least 0.2 M added NaCl and that transcription occurs in good yield to at least 0.4 M added NaCl (at these concentrations of enzyme and promoter DNA). Note the increase in some shorter RNA transcripts for the tight binding constructs, indicated in the figures with an asterisk. This has been observed previously for the pss[−5] construct and it has been suggested that strengthening promoter binding leads to a (statistically distributed) delay in promoter release, which results in the premature release of 11–13mer transcripts (16,17,21). In these results, the nicked construct behaves similarly, consistent with stronger promoter binding (18).

### Reduction of self-primed extension increases yield of encoded RNA

As noted above and previously demonstrated (26), one would expect the rebinding of product RNA that initiates self-primed extension to be salt sensitive (and independent of the promoter that drives its extension). To confirm this prediction here, we repeated the above experiment, in Figure 3, with a DNA sequence (panel A) encoding an RNA previously shown to generate self-primed extension products (10). The results confirm this prediction, as transcription from constructs that require melting (Figure 3B and C) shows high sensitivity to added NaCl, as seen in Figure 2. Transcription from constructs with eliminated/decoupled promoter melting (Figure 3D and E) again shows salt tolerance, but now with dramatically reduced self-primed extension. Note that since the 34mer RNA is chased less to extended products, the net yield of 34mer increases.

The significant net increase in the amount of the correct length product in Figure 3B and C is consistent with salt-driven inhibition of self-primed extension from the encoded product, even as initiation decreases. Strengthening promoter binding with the pss[−5] and nick[−5, −4] constructs (Figure 3D and E, respectively) further increases the yield of the encoded 34mer RNA and allows productive transcription at higher added salt concentrations.

The above results present a new opportunity for those using T7 RNA polymerase to synthesize RNA with increased yield and purity. Note that lower concentrations of enzyme and/or DNA should show slightly more salt sensitivity, while higher concentrations may extend salt tolerance beyond that shown here. Depending on the application, one can balance higher purity versus higher yield of the encoded RNA.

### Enzymatic introduction of gaps

While the above direct assembly approach will be practical for those using synthetic oligonucleotides as templates, there is a large demand for RNAs of longer lengths, where the independent synthesis of each single DNA strand is expensive or impossible. For the *in vitro* synthesis of long RNAs, it is common to generate (fully) double-stranded promoter-containing templates using the PCR.

Initially, to mimic the nick [−5, −4] construct of Figures 2 and 3, we prepared dsDNA in which the nontemplate strand of the promoter contains a dU base uniquely at position −4 of the promoter. Following duplex assembly, we then incubated the double-stranded DNA with the Thermolabile USER^®^ II Enzyme system from New England Biolabs. With this reagent kit, uracil DNA glycosylase excises the uracil base at position −4 and then endonuclease III cleaves the phosphodiester backbone on either side of the sugar, generating a single nucleotide gap at position −4.

The results shown in Figure 4, using the same sequence as that used in Figure 2, confirm the hypothesis. Only the USER II enzymatic excision of dU at position −4 (Figure 4A) of the nontemplate strand provides salt tolerance, as shown in Figure 4E. The controls presented in Figure 4B–D are all expected to remain fully double stranded and, as expected, show native salt sensitivity. Excision (or lack thereof) is confirmed in Supplementary Figure S1.

Finally, to reproduce the results of Figure 3 in this system, we resynthesized the nontemplate strand of that sequence, but now with dU at position −4. As shown in Figure 5A–C, the unreacted DNA drives transcription that is essentially the same as the results in Figure 3A–C. We then excised dU from the DNA with the Thermolabile USER^®^ II Enzyme system to create a gap at position −4. As expected, the transcription from this DNA yields the same salt tolerance and, critically, the same reduction in self-primed extension observed in Figure 3D and E. Specifically, the enzymatically created gap yields the same results as the assembled nick.

The results from the above sets of experiments clearly confirm that for the fully double-stranded system, with its native binding affinity, increasing salt concentrations decreases initiation (and self-primed extension). The pss[−5], nicked and gapped constructs all increase promoter binding and so increase the salt tolerance of initiation, while at the same time allowing salt to inhibit self-primed extension. The novel Thermolabile USER^®^ II Enzyme gapping approach allows users who cannot assemble DNA from pieces to take advantage of this substantial improvement to *in vitro* transcription.

### Extension to longer RNAs

The approach characterized above should be readily extendable to long RNAs, including CRISPR guide RNA (sgRNA), messenger RNA or long noncoding RNAs. Double-stranded DNA of arbitrary length for *in vitro* transcription is routinely generated by the PCR. By including the promoter sequence in the PCR primer, one can include a dU base (as a part of the primer) to direct excision of the base at promoter position −4, as above. Accordingly, we prepared PCR primers in which the primer corresponding to the nontemplate strand of the promoter contains a dU base uniquely at position −4 of the promoter. Following PCR and standard product cleanup, we incubated the double-stranded DNA with the Thermolabile USER^®^ II Enzyme.

As an initial demonstration, template encoding a 104-nucleotide guide RNA was PCR amplified with dU at position −4 of nontemplate, as shown in Figure 6A. To ensure replication opposite dU in the primer, PCR amplification used Phusion U DNA polymerase (Taq DNA polymerase is reported to have a higher error rate). Transcription from the constructs with dU intact or excised was challenged with increasing salt, as above. The gel analysis shown in Figure 6B confirms that the enzymatically gapped construct tolerates higher salt conditions. Note that a diffuse band migrating just above the primary band decreases in intensity with increasing salt, while the primary band increases in intensity. This is consistent with assignment of the upper band as primer-extended RNA, deriving from the primary band, mimicking the behavior seen with shorter RNAs.

### Reduced dsRNA contamination in high-yield RNA preparations from long DNA templates

The above assays intentionally used relatively short RNAs, as the analytical tools provide good to reasonable resolution at these lengths, but there is no reason that the synthesis of much longer RNAs will not benefit equally well. To confirm this expectation, a PCR amplified template with dU at position −4 and encoding a 1700-nucleotide mRNA was reacted with the USER^®^ II system and then transcribed under increasing salt concentrations, as before. The data in Figure 7A show that overall, salt sensitivity is higher, as expected in a reaction carried out at lower enzyme and promoter DNA concentrations, but the USER II-reacted construct again shows significantly higher salt tolerance relative to the unreacted control (the enzymatically gapped DNA template tolerates salt up to 200–300 mM without significant loss in yield, while the fully duplex construct shows dramatically reduced transcription at 200 mM).

At these lengths, primed extension products are expected not to be resolved. Instead, the effect of the increasing salt on dsRNA synthesis was analyzed by carrying out an immunoblot with anti-dsRNA antibody (J2 clone) that specifically binds to dsRNA longer than ∼40 bp. Immunoblot data shown in Figure 7C confirm that dsRNA is produced during transcription at lower salt conditions, while transcription with elevated salt in the transcription reaction decreases the formation of these dsRNA by-products dramatically.

### *In vivo* functionality of RNA from gapped templates

Transcribed mRNA used in therapeutics needs both a 5′ cap and a 3′ poly-A tail to be functional *in vivo* and these modifications can be added during or after transcription (27,28). To show that these modifications can be added to the gapped synthesis system and that mRNAs synthesized under the high-salt reaction conditions are functional, an mRNA template encoding the luciferase reporter with an AG transcription start site and a poly-A tail was generated with a dU forward primer and a poly-T containing reverse primer, then reacted with the USER^®^ II system and transcribed in the presence of cap analog CleanCap AG with increasing levels of added salt. As shown in Figure 8A and B, transcription of this capped mRNA also exhibits salt tolerance comparable to the transcription profiles above and shows less dsRNA by-product formation compared to lower salt transcriptions.

To determine whether the mRNA synthesized under high-salt conditions is functional and is translated *in vivo*, 1 μg of RNA was transfected into HEK 293 cells and the expression from the synthetic mRNA was measured. The results in Figure 8D clearly demonstrate that high-salt-generated mRNA yielded comparable *in vivo* expression of the luciferase and functioned similarly to HPLC-purified capped luciferase mRNA, which is a standard method to get rid of dsRNA by-products from *in vitro* transcription reactions.

Yet another illustration of this approach is provided in Supplementary Figure S9, showing synthesis of an 857-nucleotide-long mRNA of therapeutic relevance. For this reaction, we used concentrations of enzyme and DNA (0.25 μM each) that were higher than those in the transcription reactions of Figures 6–8 (but lower than those of Figures 2–5). As expected from mass action, transcription from gapped templates proceeds well even at 0.4 M added salt, as seen for the similarly driven reactions of Figures 2–5. This demonstrates that transcription conditions (salt concentrations) can be adjusted depending on the concentrations of enzyme and DNA that can be provided to the reaction.

The above results demonstrate the utility of this overall approach in the synthesis of very short RNAs, sgRNAs and long mRNAs with and without cotranscriptional 5′ cap incorporation and an encoded poly-A tail.

## DISCUSSION

Production of high yields of RNA free from double-stranded self-primed extension impurities is critical to the broad success of mRNA therapeutics, as well as to RNA research more broadly. Extended RNA products arise primarily through rebinding of product RNA to the polymerase (8–10) and we have recently shown that high salt concentrations reduce or eliminate this rebinding (26). Unfortunately, high salt also inhibits promoter binding that leads to (correct) transcription initiation [but does not inhibit elongation, since elongation complex stability is conferred by topological locking of the RNA around the DNA (29)]. We have demonstrated that tethering the polymerase to the promoter increases local relative concentrations (effectively strengthening binding) and allows transcription at higher salt (26). The simpler approach to allowing high-salt transcription described here will be more broadly useful to the wide RNA community.

In this study, we initially exploit a previously characterized modification to the promoter DNA that has been shown to strengthen promoter binding significantly. A well-established model for promoter binding by T7 RNA polymerase argues that (favorable) protein contacts with the duplex promoter DNA upstream of and including position −5 drive binding and that some of this binding energy is used to drive (unfavorable) local melting of the promoter from position −4 to ∼+3 (18,19,30–33). Thus, the commonly used construct pss[−5] that is single stranded from position −4 downstream has been shown to bind more strongly to the polymerase (18).

Combining high-salt transcription with the more tightly binding pss[−5] promoter variant does yield a dramatic reduction in self-primed extensions, while maintaining high yields of the encoded RNA, as demonstrated in Figures 2D and 3D. Under these conditions (but see below), transcription at 0.2–0.4 mM added NaCl provides a substantial increase in purity of the desired product, reducing or eliminating the need for extensive post-purification of the product RNA, while maintaining good yield. End users of this approach can empirically choose to balance yield and purity, as needed.

Consistent with the mechanistic model, a construct that is double stranded in the melting region, but is single stranded *beyond* the melting region (pss[+2]) shows the high salt sensitivity of the fully double-stranded construct, as shown in Figures 2C and 3C. Promoter-driven melting near the start site involves binding-directed intercalation of the protein ‘Val loop’ into the DNA duplex between positions −5 and −4 (32,33). This model predicts that instead of removing the barrier, one could reduce the barrier to intercalation by introducing a break (‘nick’) in the DNA between positions −5 and −4 of the nontemplate strand, thereby strengthening promoter binding (the break will also lead to enhanced ‘breathing’ of DNA in the melting region, lowering the barrier to melting that is required for *de novo* initiation). Assembling such a nicked construct by combining the three DNA fragments yields the expected behavior, as illustrated in Figures 2E and 3E.

Assembly of a nicked promoter DNA from fragments will work well for those transcribing up to ∼50–70-nucleotide RNAs but transcribing a 70-nucleotide RNA requires a DNA template strand of ∼90 nucleotides, and at these lengths, the yields and purity of chemically synthesized DNA are low. With an eye toward generating longer RNAs, we can replace the T at position −4 of the nontemplate strand with a dU and then target that position with the USER^®^ II Enzyme system to generate a single nucleotide ‘gap’ at position −4, functionally substituting for the nick between positions −5 and −4. Paralleling the results in Figures 2 and 3, the data presented in Figures 4 and 5 confirm the predictions. The substitution of T by dU alone yields no change in behavior, but excising the dU to create a gap yields salt-tolerant transcription and increasing salt concentrations reduces primed extension, as above.

Of course, the potential utility of this new approach derives from the ability to generate DNA templates (incorporating a dU at position −4 of the promoter through the upstream PCR primer) encoding arbitrarily long RNAs. The results presented in Figure 6 demonstrate the enzymatic synthesis of a 104-nucleotide guide RNA using this new approach. In this case, RNA synthesized in the presence of no added salt includes a more slowly migrating band, which decreases with increasing salt, consistent with its deriving from primed extension. As before, in the gapped, but not the native, system, transcription proceeds well to 0.2 M added NaCl.

Moving to significantly longer RNAs, we next characterized the synthesis of a 1700-nucleotide mRNA. The Bioanalyzer results in Figure 7A again show the characteristic increase in salt tolerance of transcription from the gapped promoter DNA. Note that as longer RNAs are synthesized, primer-extended products are expected to become broader in their length distribution, while electrophoretic approaches are poorly resolving. To ask whether dsRNA products are being generated under normal conditions and whether this new approach reduces them, we utilized immunoblots to detect dsRNA. The results in Figure 7B, in which equal amounts of total RNA were probed with antibodies against dsRNA, confirm the presence of dsRNA under standard (0 M added NaCl) conditions, which is reduced substantially for transcription in the presence of increasing salt.

To further demonstrate the generalizability of the approach and to introduce another analytical assay, a luciferase mRNA (M3) was synthesized incorporating a 5′ cap and encoding a poly-A tail (1821 nucleotides in length). The results shown in Figure 8 are very similar to those in Figure 7. Figure 8A demonstrates the increased salt tolerance, while the immunoblot data in Figure 8C confirm a reduction in contaminating dsRNA. Finally, functionality of the mRNA was demonstrated by a functional luminescence assay in Figure 8D.

The dramatic reduction of the dsRNA by-products in the poly-A tail containing mRNAs confirms the hypothesis that during *in vitro* transcription, RNAs are also extended by a templated, self-priming mechanism by the single subunit RNA polymerases such as T7. While a priming stem containing As ‘paired’ with As is predicted to be unstable, addition of a single U to the 3′ end creates a priming structure that is more stable. Addition of a second and a third U would then generate good complementarity to prime extension. Alternatively, the poly-A tail may loop back (over a long distance in this case) to prime in the mixed nucleotide region of the RNA. Future studies will distinguish between these two mechanisms, but the utility of high-salt synthesis from gapped promoter constructs is unambiguously clear.

## CONCLUSION

The results presented here demonstrate the broad utility of synthesizing RNA under elevated salt concentrations using promoter DNA engineered to have increased binding affinity for the polymerase. Increased salt reduces rebinding of ‘good’ product RNA, preventing it from being converted into undesired dsRNA impurity, while the increased promoter binding allows good yields under the higher salt conditions.

In this system, there is predictable interplay between salt sensitivity and the concentrations of the DNA and the RNA polymerase. End users will want to empirically determine the ideal concentration of added salt, to balance this and to balance trade-offs of yield versus purity. Higher concentrations of DNA and RNA polymerase (and longer reaction times) may also lead to increased concentrations of product RNA, necessitating higher salt to prevent/limit rebinding.

While those synthesizing short RNAs (in other words, using promoter DNA assembled from chemically synthesized DNA oligonucleotides) can readily adapt their protocols to high salt, longer RNAs can be synthesized from PCR-generated DNA, with a fairly simple and inexpensive adaptation in the PCR. For ‘worst case’ sequences, this can yield a much higher total yield of the target RNA. For all RNAs, the RNA should be of higher purity, with reduced or eliminated levels of contaminating RNAs derived from self-primed extension of the RNA.

## DATA AVAILABILITY

The data underlying this article will be shared on reasonable request to the corresponding author.

## Supplementary Material

gkad027_Supplemental_File
