# Supplementary material for: High-salt transcription from enzymatically gapped promoters nets higher yields and purity of transcribed RNAs
Source: Nucleic Acids Res. 2023 Jan 31;51(6):e36. doi: 10.1093/nar/gkad027 (PMC10085681; doi:10.1093/nar/gkad027)
Supplement: gkad027_Supplemental_File [file gkad027_supplemental_file.pdf]

Supplementary Material

High Salt Transcription from Enzymatically Gapped Promoters Nets Higher Yields and Purity of Transcribed RNAs

Kithmie MalagodaPathiranage[1], Elvan Cavac[1], Tien-Hao Chen[§], Bijoyita Roy[§], and Craig T. Martin[1,*]

TABLE SI

DNAs labelled 34Alt below were used in Figures 2 and 4. DNAs labelled 34sl were used in Figures 3 and 5. DNAs labelled sgRNA104 below was used in Figure 6. DNAs labelled M2 below was used in Figure 7. DNAs labelled M3 below was used in Figure 8. DNAs labelled M4 below was used in Figure S2.

DNA	sequences
Nontemplate [-20, +2]	5'-AATTAATACGACTCACTATAGG-3'
Nontemplate [-20, -5]	5'-AATTAATACGACTCAC-3'
Nontemplate 34alt[-4,+34]	5'-TATAGGAGGTATGACTCGAAACTACGTCGACGCATTTA-3'
Nontemplate 34sl [-4, +34]	5'-TATAGGAGGTACGACTCGCACGTAGAGGTGAAGATTTA-3'
Template 34sl	3'-TTAATTATGCTGAGTGATATCCTCCATGCTGAGCGTGCATCTCCACTTCTAAAT-5'
Template 34alt	3'-TTAATTATGCTGAGTGATATCCTCCATACTGAGCTTTGATGCAGCTGCGTAAAT-5'
NT 34sl [-20, +34]	5'-AATTAATACGACTCAC**u**ATAGGAGGTACGACTCGCACGTAGAGGTGAAGATTTA-3'
NT 34alt [-20, +34]	5'-AATTAATACGACTCAC**u**ATAGGAGGTATGACTCGAAACTACGTCGACGCATTTA-3'
U NT 34sl [-20, +34]	5'-AATTAATACGACTCAC**u**ATAGGAGGTACGACTCGCACGTAGAGGTGAAGATTTA-3'
U NT 34alt [-20, +34]	5'-AATTAATACGACTCAC**u**ATAGGAGGTATGACTCGAAACTACGTCGACGCATTTA-3'
sg RNA 104 (nontemplate strand shown)	5'-AATTAATACGACTCAC**u**ATAGGAGGAAATTAATACGACTCACTATGTTTTAGAGCTAGAAATA<br>GCAGTTAAAATAAGGCTAGTCCGTTATCAACTTGAAAAAGTGGCACCGAGTCGGTGCTTTT-3'
M2 (nontemplate strand shown)	5'-TTAATACGACTCAC**u**ATAGGTCTAGAAATAATTTTGTTTAACTTTAAGAAGGAGATATAACCA<br>TGAAAATCGAAGAAGGTAAAGGTCACCATCACCATCACCACGGATCCATGGAAGACGCCAAAAACA<br>TAAAGAAAGGCCCGGCGCCATTCTATCCTCTAGAGGATGGAACCGCTGGAGAGCAACTGCATAAGG<br>CTATGAAGAGATACGCCCTGGTTCCTGGAACAATTGCTTTTACAGATGCACATATCGAGGTGAACA<br>TCACGTACGCGGAATACTTCGAAATGTCCGTTCGGTTGGCAGAAGCTATGAAACGATATGGGCTGA<br>ATACAAATCACAGAATCGTCGTATGCAGTGAAAACTCTCTTCAATTCTTTATGCCGGTGTTGGGCG<br>CGTTATTTATCGGAGTTGCAGTTGCGCCCGCGAACGACATTTATAATGAACGTGAATTGCTCAACA<br>GTATGAACATTTCGCAGCCTACCGTAGTGTTTGTTTCCAAAAAGGGGTTGCAAAAAATTTTGAACG<br>TGCAAAAAAAATTACCAATAATCCAGAAAATTATTATCATGGATTCTAAAACGGATTACCAGGGAT<br>TTCAGTCGATGTACACGTTCGTCACATCTCATCTACCTCCCGGTTTTAATGAATACGATTTTGTAC<br>CAGAGTCCTTTGATCGTGACAAAACAATTGCACTGATAATGAATTCCTCTGGATCTACTGGGTTAC<br>CTAAGGGTGTGGCCCTTCCGCATAGAACTGCCTGCGTCAGATTCTCGCATGCCAGAGATCCTATTT<br>TTGGCAATCAAATCATTCCGGATACTGCGATTTTAAGTGTTGTTCCATTCCATCACGGTTTTGGAA<br>TGTTTACTACACTCGGATATTTGATATGTGGATTTCGAGTCGTCTTAATGTATAGATTTGAAGAAG<br>AGCTGTTTTTACGATCCCTTCAGGATTACAAAATTCAAAGTGCGTTGCTAGTACCAACCCTATTTT<br>CATTCTTCGCCAAAAGCACTCTGATTGACAAATACGATTTATCTAATTTACACGAAATTGCTTCTG<br>GGGGCGCACCTCTTTCGAAAGAAGTCGGGGAAGCGGTTGCAAAACGCTTCCATCTTCCAGGGATAC<br>GACAAGGATATGGGCTCACTGAGACTACATCAGCTATTCTGATTACACCCGAGGGGGATGATAAAC<br>CGGGCGCGGTCGGTAAAGTTGTTCCATTTTTTGAAGCGAAGGTTGTGGATCTGGATACCGGGAAAA<br>CGCTGGGCGTTAATCAGAGAGGCGAATTATGTGTCAGAGGACCTATGATTATGTCCGGTTATGTAA<br>ACAATCCGGAAGCGACCAACGCCTTGATTGACAAGGATGGATGGCTACATTCTGGAGACATAGCTT<br>ACTGGGACGAAGACGAACACTTCTTCATAGTTGACCGCTTGAAGTCTTTAATTAAATACAAAGGAT<br>ATCAGGTGGCCCCCGCTGAATTGGAATCGATATTGTTACAACACCCCAACATCTTCGACGCGGGCG

	TGGCAGGTCTTCCCGACGATGACGCCGGTGAACTTCCCGCCGCCGTTGTTGTTTTGGAGCACGGAAAGACGATGACGGAAAAAGAGATCGTGGATTACGTCGCCAGTCAAGTAACAACCGCGAAAAAGTTGCGCGGAGGAGTTGTGTTTGTGGACGAAGTACCGAAAGGTCTTACCGGAAAACTCGACGCAAGAAAAATCAGAGAGATCCTCATAAAGGCCAAGAAGGGCGGAAAGTCCAAACTCGAGTAAGGTT-3’
M3 (nontemplate strand shown)	5’-TAATACGACTCAC**u**ATAAGGAGACCCAAGCTTGGTACCGAGCTCGGATCCGCCACCATGAAGACCTTAATTCTTGCCGTTGCATTAGTCTACTGCGCCACTGTTCATTGCCAGGACTGTCCTTACGAACCTGATCCACCAAACACAGTTCCAACTTCCTGTGAAGCTAAAGAAGGAGAATGTATTGATAGCAGCTGTGGCACCTGCACGAGAGACATACTATCAGATGGACTGTGTGAAAATAAACCAGGAAAAACATGTTGCCGAATGTGTCAGTATGTAATTGAATGCAGAGTAGAGGCCGCAGGATGGTTTAGAACATTCTATGGAAAGAGATTCCAGTTCCAGGAACCTGGTACATACGTGTTGGGTCAAGGAACCAAGGGCGGCGACTGGAAGGTGTCCATCACCCTGGAGAACCTGGATGGAACCAAGGGGGCTGTGCTGACCAAGACAAGACTGGAAGTGGCTGGAGACATCATTGACATCGCTCAAGCTACTGAGAATCCCATCACTGTAAACGGTGGAGCTGACCCTATCATCGCCAACCCGTACACCATCGGCGAGGTCACCATCGCTGTTGTTGAGATGCCAGGCTTCAACATCACCGTCATTGAGTTCTTCAAACTGATCGTGATCGACATCCTCGGAGGAAGATCTGTAAGAATCGCCCCAGACACAGCAAACAAAGGAATGATCTCTGGCCTCTGTGGAGATCTTAAAATGATGGAAGATACAGACTTCACTTCAGATCCAGAACAACTCGCTATTCAGCCTAAGATCAACCAGGAGTTTGACGGTTGTCCACTCTATGGAAATCCTGATGACGTTGCATACTGCAAAGGTCTTCTGGAGCCGTACAAGGACAGCTGCCGCAACCCCATCAACTTCTACTACTACACCATCTCCTGCGCCTTCGCCCGCTGTATGGGTGGAGACGAGCGAGCCTCACACGTGCTGCTTGACTACAGGGAGACGTGCGCTGCTCCCGAAACTAGAGGAACCTGCGTTTTGTCTGGACATACTTTCTACGATACATTTGACAAAGCAAGATACCAATTCCAGGGTCCCTGCAAGGAGATTCTTATGGCCGCCGACTGTTTCTGGAACACTTGGGATGTGAAGGTTTCACACAGGAATGTTGACTCTTACACTGAAGTAGAGAAAGTACGAATCAGGAAACAATCGACTGTAGTAGAACTCATTGTTGATGGAAAACAGATTCTGGTTGGAGGAGAAGCCGTGTCCGTCCCGTACAGCTCTCAGAACACTTCCATCTACTGGCAAGATGGTGACATACTGACTACAGCCATCCTACCTGAAGCTCTGGTGGTCAAGTTCAACTTCAAGCAACTGCTCGTCGTACATATTAGAGATCCATTCGATGGTAAGACTTGCGGTATTTGCGGTAACTACAACCAGGATTTCAGTGATGATTCTTTTGATGCTGAAGGAGCCTGTGATCTGACCCCCAACCCACCGGGATGCACCGAAGAACAGAAACCTGAAGCTGAACGACTCTGCAATAGTCTCTTCGCCGGTCAAAGTGATCTTGATCAGAAATGTAACGTGTGCCACAAGCCTGACCGTGTCGAACGATGCATGTACGAGTATTGCCTGAGGGGACAACAGGGTTTCTGTGACCACGCATGGGAGTTCAAGAAAGAATGCTACATAAAGCATGGAGACACCCTAGAAGTACCAGATGAATGCAAATAGGCGGCCGCAATAAAATATCTTTATTTTCATTACATCTGTGTGTTGGTTTTTTGTGTGTCTAAAAAAAAAAAAAAAAAAAAAAAAAAAAAAAAAAAAAAAAAAAAAAAAAAAAAA-3’
M4 (nontemplate strand shown)	5’-TCGAAATTAATACGACTCAC**u**ATAAGGGCTTGCTTGTTCTTTTTGCAGAAGCTCAGAATAAACGCTCAACTTTGGCACCATGGGAGTGCACGAGTGTCCCGCGTGGTTGTGGTTGCTGCTGTCGCTCTTGAGCCTCCCACTGGGACTGCCTGTGCTGGGGGCACCACCCAGATTGATCTGCGACTCACGGGTACTTGAGAGGTACCTTCTTGAAGCCAAAGAAGCCGAAAACATCACAACCGGATGCGCCGAGCACTGCTCCCTCAATGAGAACATTACTGTACCGGATACAAAGGTCAATTTCTATGCATGGAAGAGAATGGAAGTAGGACAGCAGGCCGTCGAAGTGTGGCAGGGGCTCGCGCTTTTGTCGGAGGCGGTGTTGCGGGGTCAGGCCCTCCTCGTCAACTCATCACAGCCGTGGGAGCCCCTCCAACTTCATGTCGATAAAGCGGTGTCGGGGCTCCGCAGCTTGACGACGTTGCTTCGGGCTCTGGGCGCACAAAAGGAGGCTATTTCGCCGCCTGACGCGGCCTCCGCGGCACCCCTCCGAACGATCACCGCGGACACGTTTAGGAAGCTTTTTAGAGTGTACAGCAATTTCCTCCGCGGAAAGCTGAAATTGTATACTGGTGAAGCGTGTAGGACAGGGGATCGCTAGGACTGACTAGGATCTGGTTACCACTAAACCAGCCTCAAGAACACCCGAATGGAGTCTCTAAGCTACATAATACCAACTTACACTTTACAAAATGTTGTCCCCCAAAATGTAGCCATTCGTATCTGCTCCTAATAAAAAGAAAGTTTCTTCACATTCTAGTAAAAAAAAAAAAAAAAAAAAAAAAAAAAAAAAAAAAAAAAAAAAAAAAAAAAAAAAAAA-3’

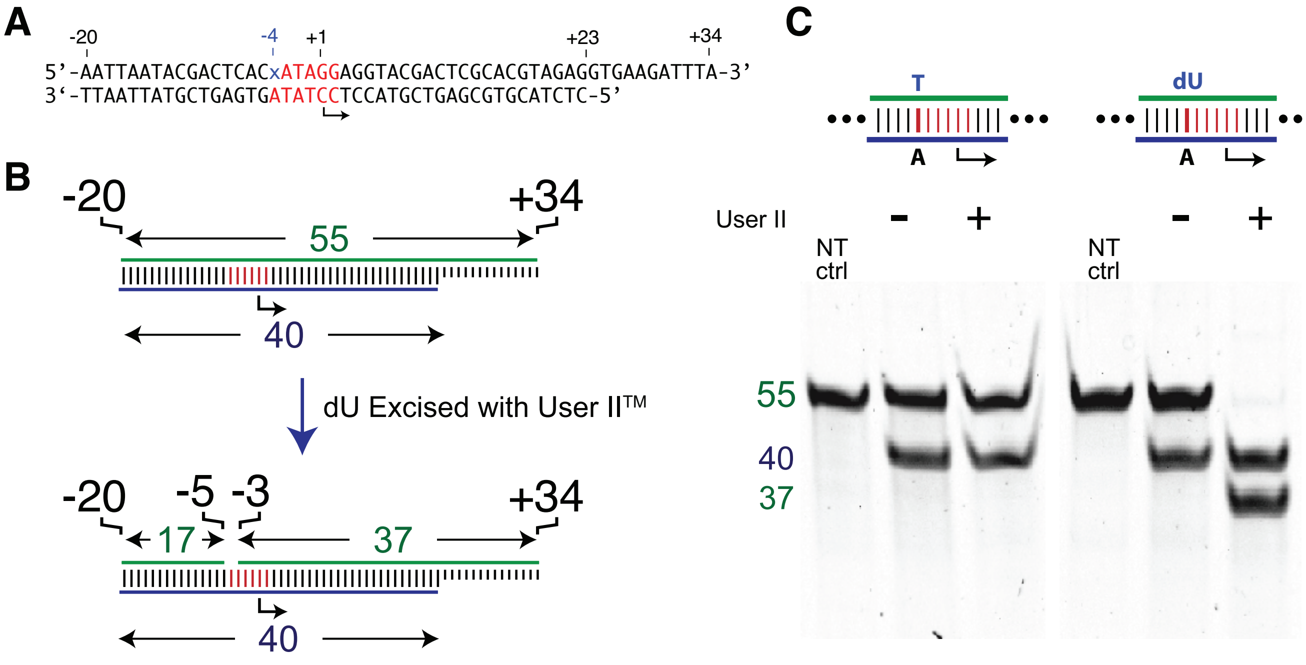

Figure S1. Thermolabile USER® II Enzyme (New England Biolabs) system shows 100% excision of double stranded DNA with dU at position -4 of the promoter. A) Assembly of nontemplate DNA with dU at -4 position with a shorter template strand allows a simple gel assay. B) Thermolabile USER® II system excision of the construct in (A) yields fragments of characteristic lengths. C) 20% acrylamide denaturing gel confirms complete excision of the dU containing nontemplate DNA (note that the 17 base fragment ran off the gel). Excision reactions were carried out with 10 μM DNA constructs at 37° C for 1 h, under conditions described in Methods.

Figures S2 to S9 following present replicate data for manuscript figures 2 to 9, respectively. In the quantitative tracings below each set of gels, horizontal alignment was adjusted slightly to correct for gel imperfections (see the gels for positioning). Since intensities of electrophoretic bands between experiments varies (radioactive decay, for example), sets of gel traces were adjusted in intensity (but intensities were not adjusted within an experimental set).

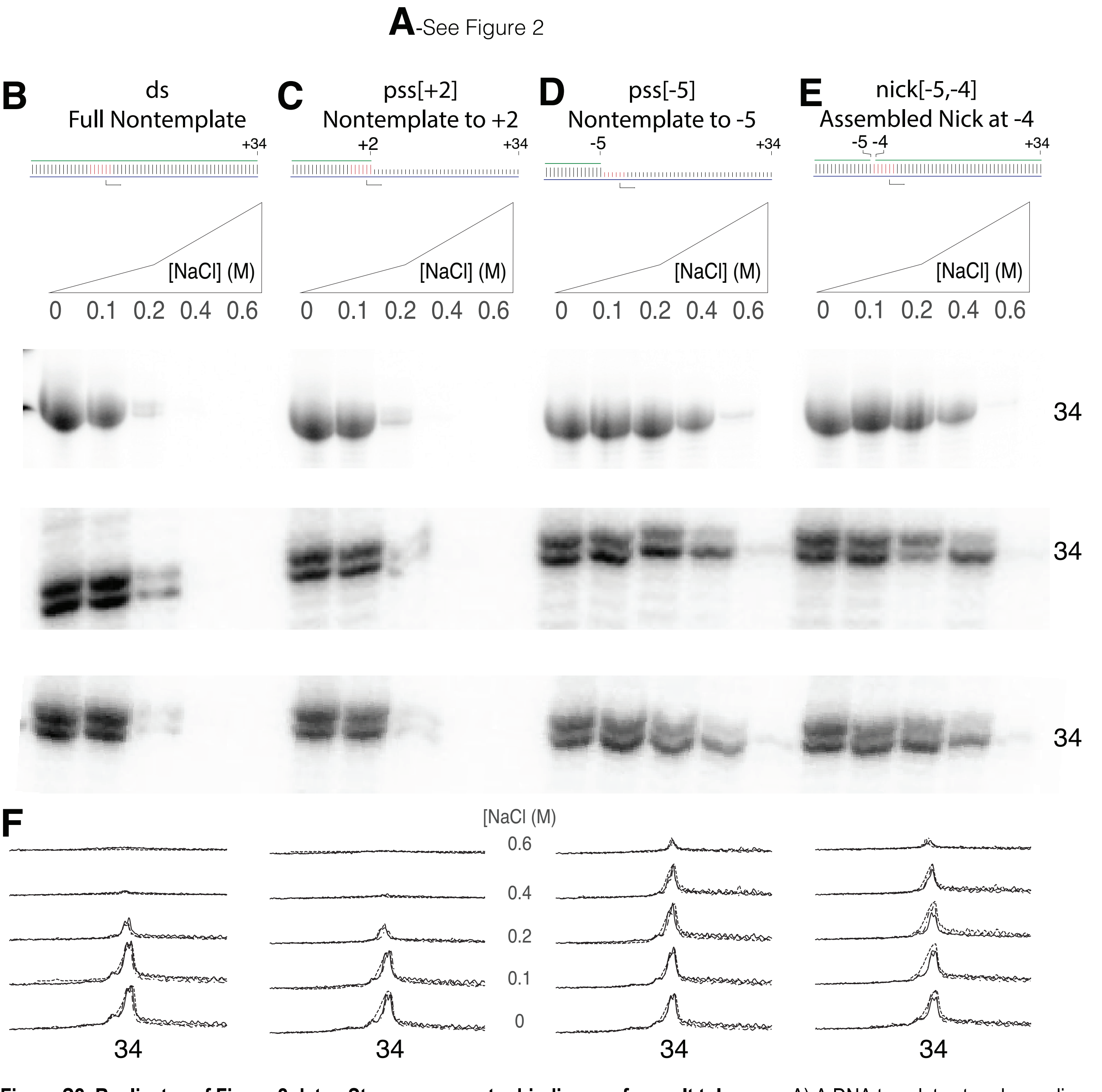

Figure S2. Replicates of Figure 2 data - Stronger promoter binding confers salt tolerance A) A DNA template strand encoding a runoff 34mer RNA known to produce relatively small amounts of self-primed extension was paired with different nontemplate DNA strands to generate constructs with native melting requirements ((B) ds and (C) pss[+2]) and relaxed melting requirements ((D) pss[-5] and (E) nick[-5,-4]). Experiments in B-E drive high yield synthesis under conditions of increasing added NaCl, as shown. Transcription reactions contained 1.0 μM DNA and 1.0 μM RNA polymerase and were incubated at 37° C for 4 h, under conditions described in Methods. The individual lane tracings in (F) quantify retention of transcription at high added salt for the relaxed melting constructs.

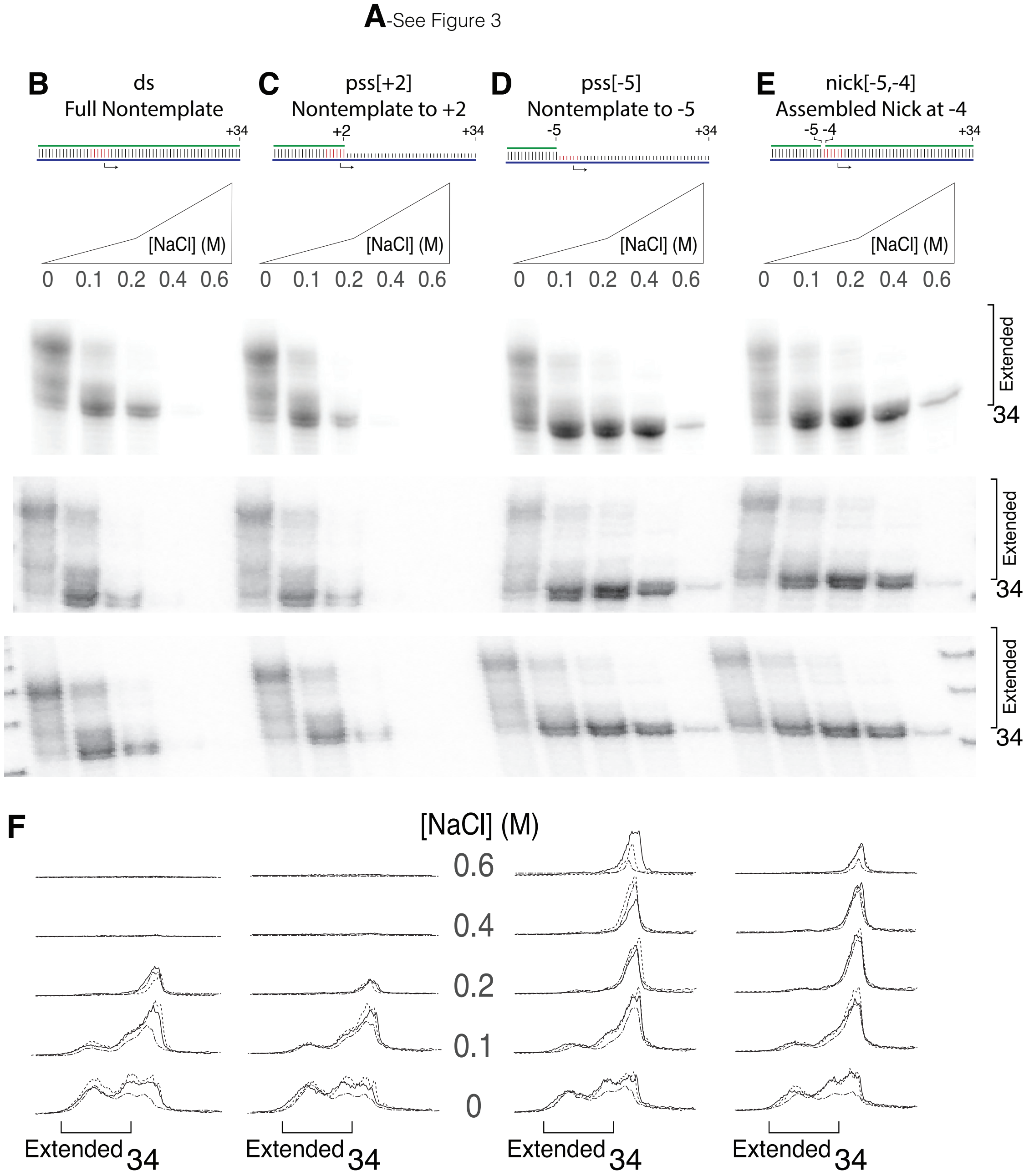

Figure S3. Replicates of Figure 3 data. Added NaCl reduces self-primed extension products. A) Template DNA encoding a runoff 34mer RNA known to prime extension was paired with nontemplate DNA strands (B-E) and transcribed as in Figure 2. The tracings in (F) quantify retention of transcription at high added salt for the relaxed melting constructs. Reactions conditions were as in Figure 2.

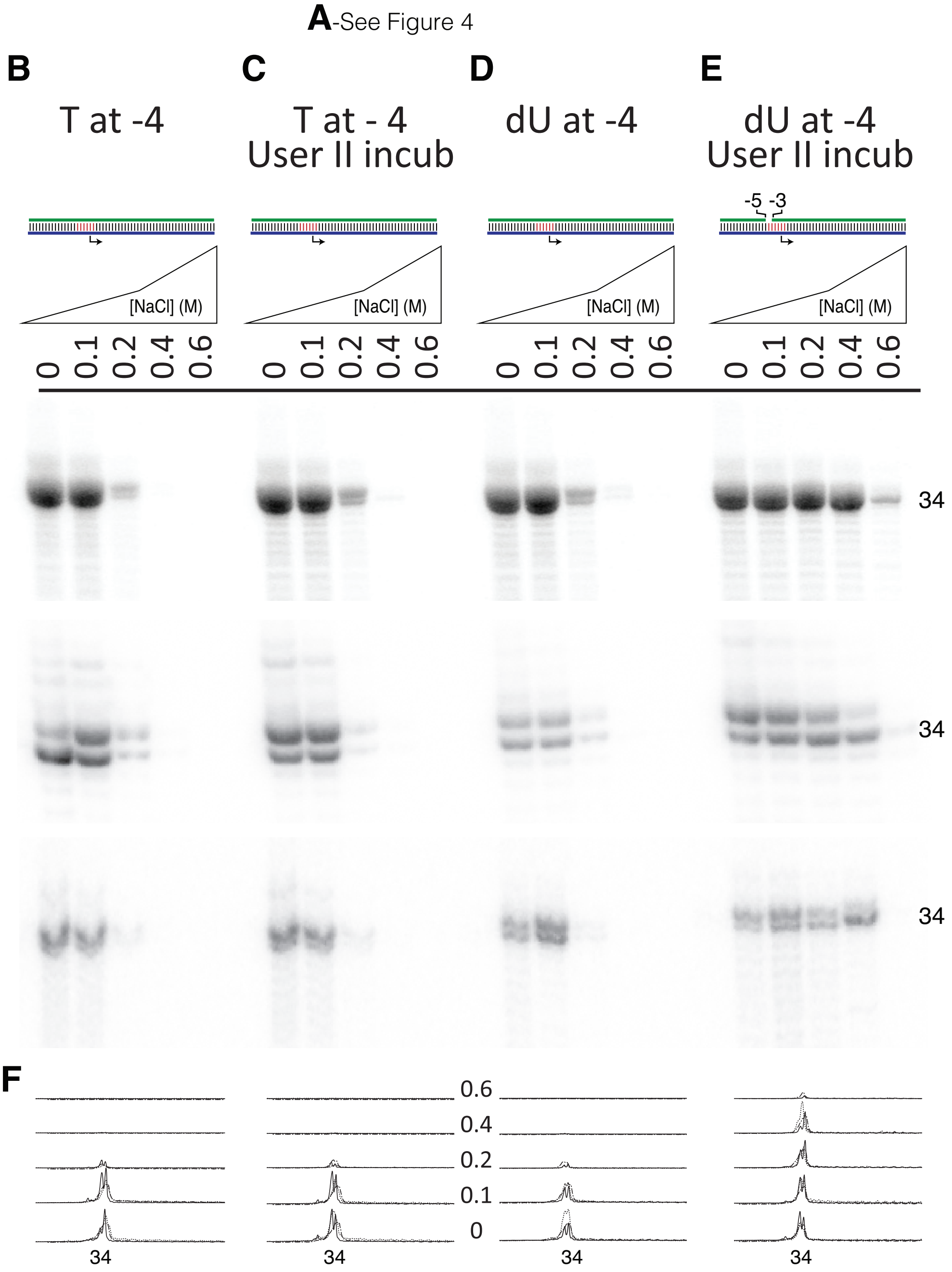

Figure S4. Replicates of Figure 4 data. Targeted enzymatic gapping increases salt tolerance. A) Template DNA encoding the runoff 34mer RNA of Figure 2 was paired with different nontemplate DNA strands: (B) and (C) have a native T at position -4 of the nontemplate strand, while (D) and (E) have dU at that position. Transcription reactions contained 1.0 μM DNA and 1.0 μM RNA polymerase and were incubated at 37° C for 4 h, under conditions described in Methods. As expected, only the enzymatic excision of dU from position -4 of the nontemplate strand (E) yields salt tolerance. Individual lane tracings are shown in (F)..

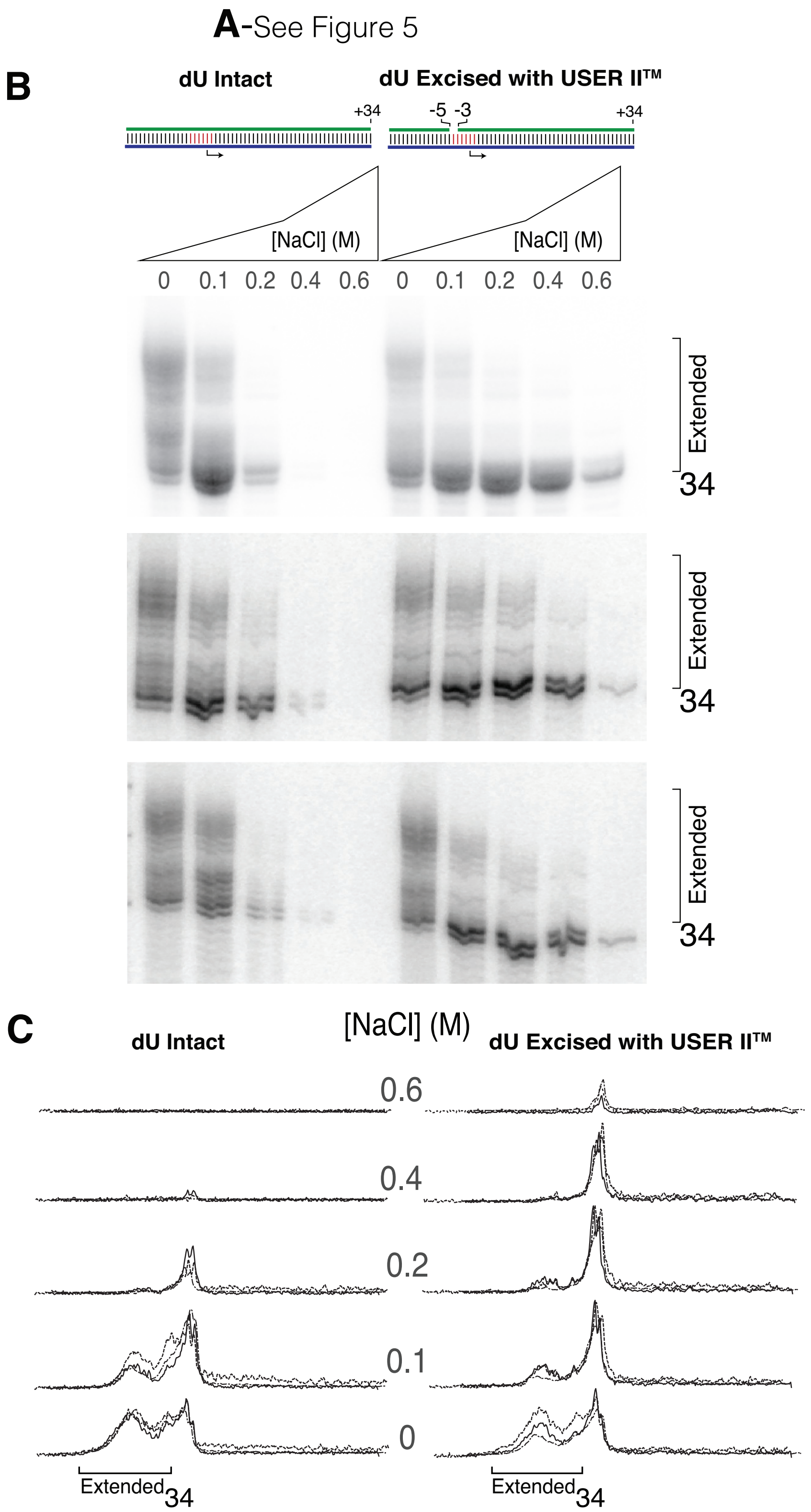

Figure S5. Replicates of Figure 5 data. A targeted gap plus high salt inhibition of self-primed extension. A) Doubled stranded DNA encoding the runoff 34mer RNA from Figure 3 and containing dU at position -4 was transcribed directly (unexcised) or following excision of dU with the USER II™ enzyme to great a gap. B) Transcription reactions contained 1.0 µM DNA and 1.0 µM RNA polymerase and were incubated at 37° C for 4 h, under conditions described in Methods. Quantification (C) demonstrates results similar to those of Figure 3. Reactions conditions were as in Figure 2.

A-See Figure 6

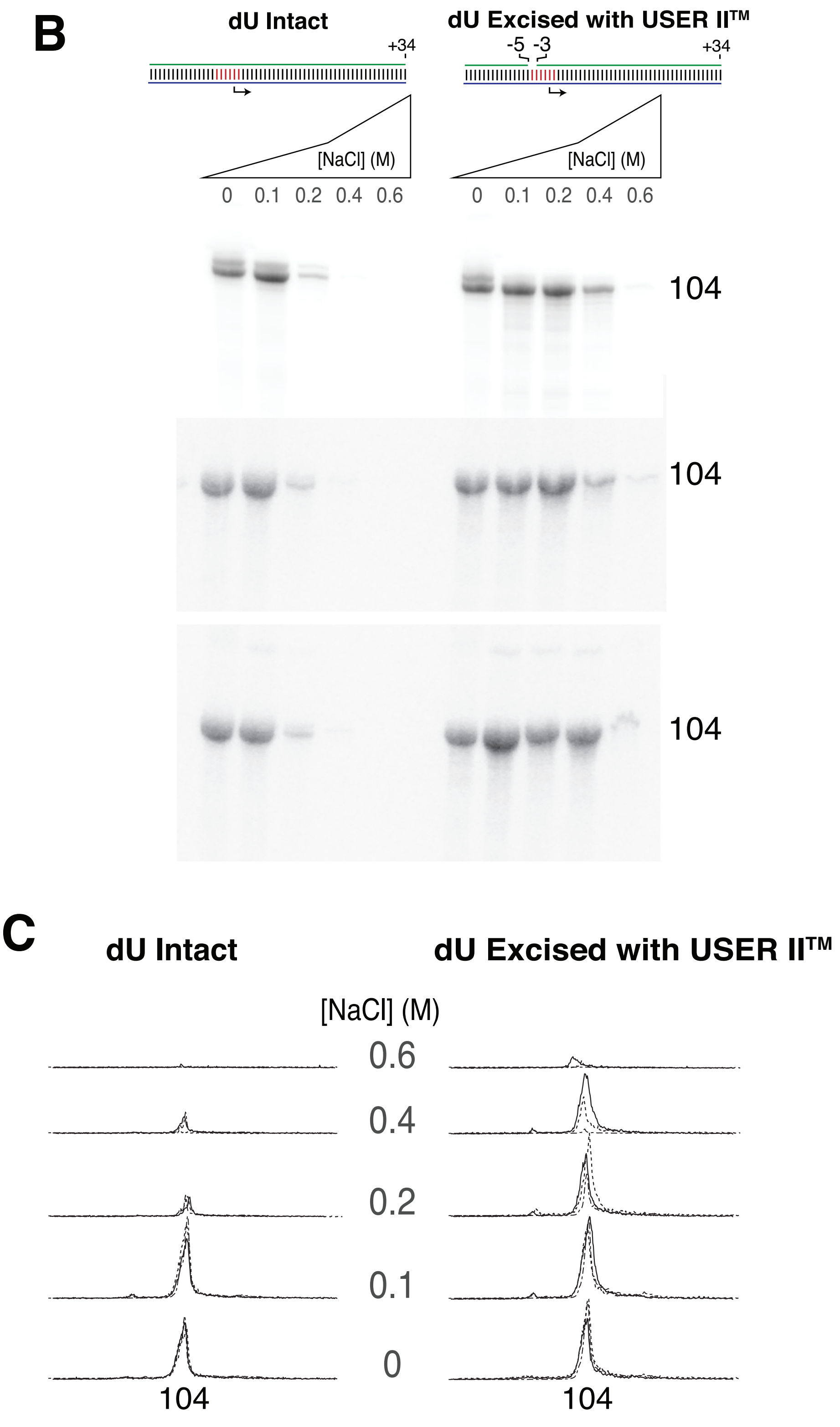

Figure S6. Replicates of Figure 6 data. Extension to a 104 nucleotide sgRNA. A) Schematic diagram of template preparation of DNA template with PCR, incorporating dU via the upstream PCR primer. B) synthesis of a 104 base guide RNA from DNA containing intact or excised dU at position -4, under elevated salt concentrations. Transcription reactions contained 0.125 µM DNA and 0.125 µM RNA polymerase and were incubated at 37° C for 4 h, under conditions described in Methods. The tracings in (C) quantify retention of transcription at high added salt.

A-See Figure 7

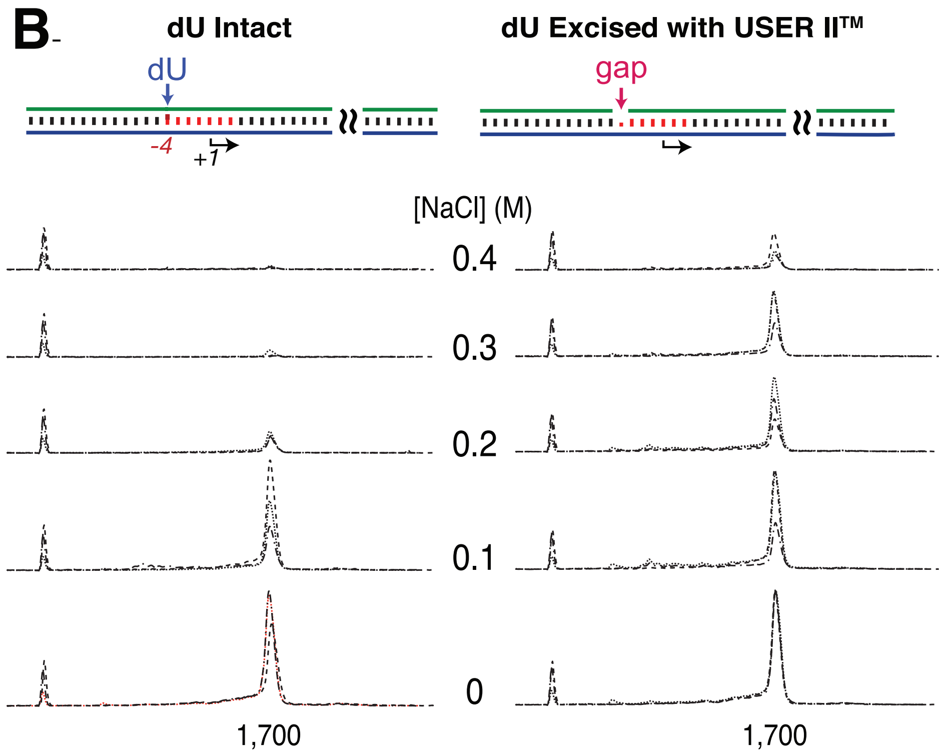

Figure S7. Replicates of Figure 7 data. Extension to 1,700 nucleotide mRNA. A) synthesis of a 1700 base mRNA from DNA containing intact or excised dU at position -4, under elevated salt concentrations. Transcription reactions contained 0.035 μM (M2) DNA and 0.035 μM RNA polymerase and were incubated at 37° C for 1 h, under conditions described in Methods. The tracings in (B) quantify retention of transcription at high added salt.

A-See Figure 8

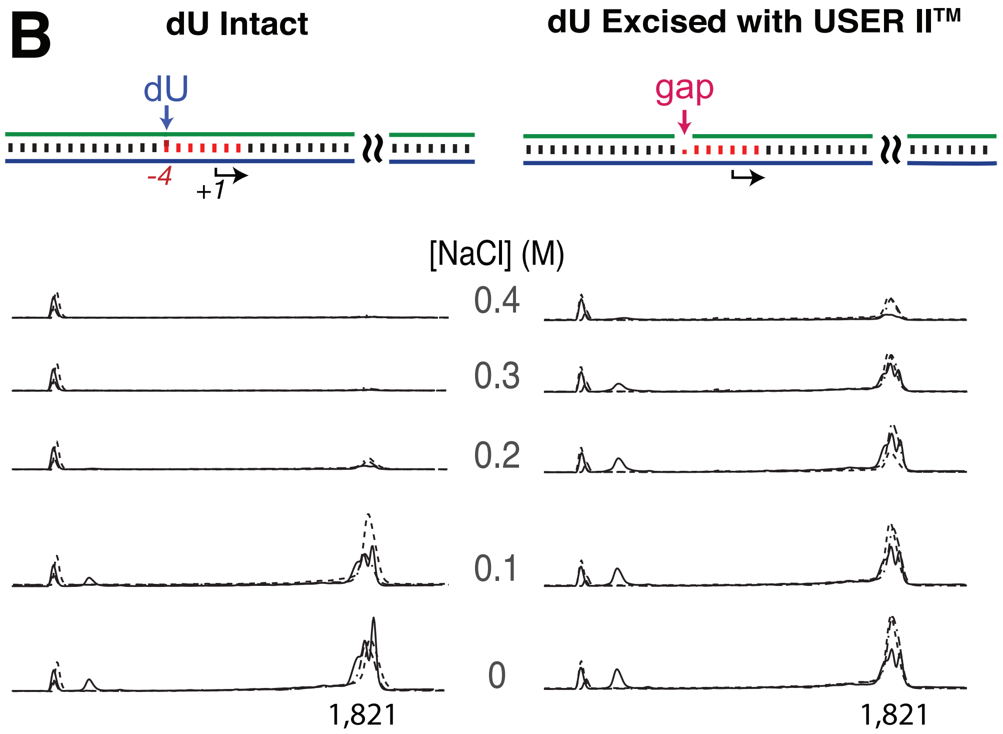

Figure S8. Replicates of Figure 8 data. mRNA generated from the gapped system is low immunogenic and functional. **(A)**Transcription of a (M3) DNA, incorporating CleanCap AG (TriLink) and encoding a poly-A tail, using DNA templates containing intact or excised dU, to generate a gap at position -4 of the promoter, as indicated. Transcription reactions contained 0.035 μM (M3) DNA and 0.035 μM RNA polymerase and were incubated at 37° C for 1 h, as described in Methods. The tracings in (B) quantify retention of transcription at high added salt.

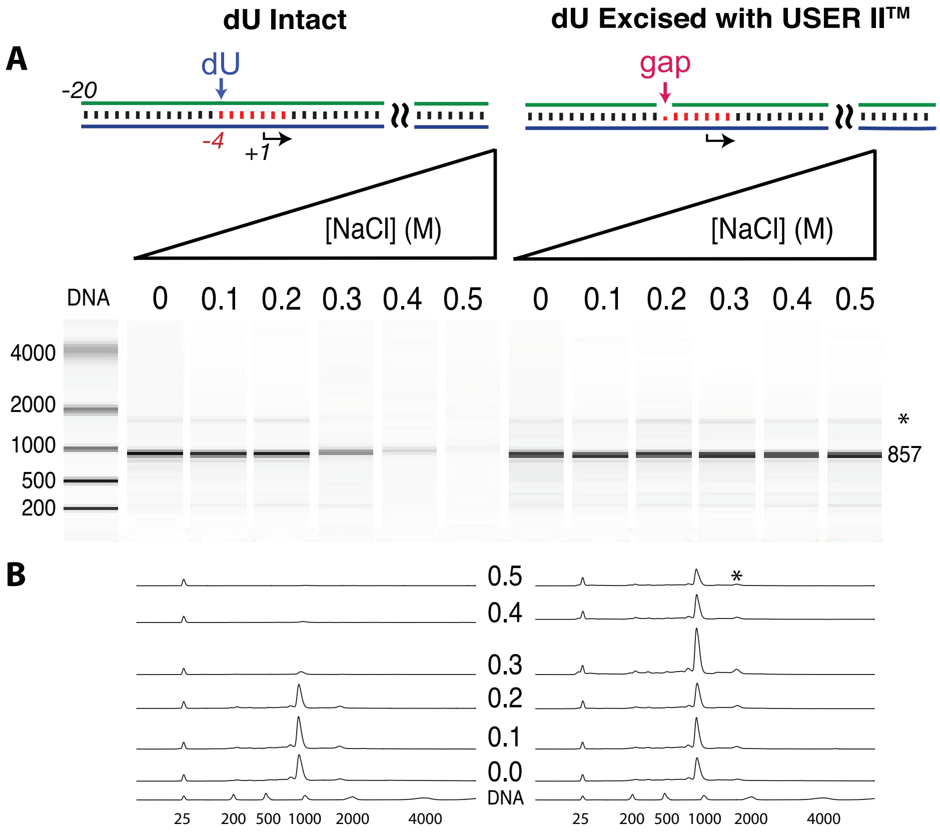

Figure S9. **Higher enzyme and DNA concentrations increase the salt tolerance of constructs during transcription**. A) A) Transcription of a (M4) DNA, incorporating CleanCap AG (TriLink) and encoding a poly-A tail, using DNA templates containing intact or excised dU, to generate a gap at position -4 of the promoter, as indicated. Transcription reactions contained 0.25 µM (M4) DNA and 0.25 µM RNA polymerase and were incubated at 37° C for 1 h, under conditions described in Methods. The Bioanalyzer primary traces in (B) quantify retention of transcription at high added salt using intact or dU-excised DNA constructs. * Structured RNA (not double stranded)
